# Translational Upregulation of an Individual p21^Cip1^ Transcript Variant by GCN2 Regulates Cell Proliferation and Survival under Nutrient Stress

**DOI:** 10.1371/journal.pgen.1005212

**Published:** 2015-06-23

**Authors:** Stacey L. Lehman, George J. Cerniglia, Gregg J. Johannes, Jiangbin Ye, Sandra Ryeom, Constantinos Koumenis

**Affiliations:** 1 Department of Radiation Oncology, Perelman School of Medicine at the University of Pennsylvania, Philadelphia, Pennsylvania, United States of America; 2 Department of Pathology and Laboratory Medicine, Drexel University College of Medicine, Philadelphia, Pennsylvania, United States of America; 3 Department of Cancer Biology, Perelman School of Medicine at the University of Pennsylvania, Philadelphia, Pennsylvania, United States of America; Fred Hutchinson Cancer Research Center, UNITED STATES

## Abstract

Multiple transcripts encode for the cell cycle inhibitor p21^Cip1^. These transcripts produce identical proteins but differ in their 5’ untranslated regions (UTRs). Although several stresses that induce p21 have been characterized, the mechanisms regulating the individual transcript variants and their functional significance are unknown. Here we demonstrate through ^35^S labeling, luciferase reporter assays, and polysome transcript profiling that activation of the Integrated Stress Response (ISR) kinase GCN2 selectively upregulates the translation of a p21 transcript variant containing 5’ upstream open reading frames (uORFs) through phosphorylation of the eukaryotic translation initiation factor eIF2α. Mutational analysis reveals that the uORFs suppress translation under basal conditions, but promote translation under stress. Functionally, ablation of p21 ameliorates G_1_/S arrest and reduces cell survival in response to GCN2 activation. These findings uncover a novel mechanism of p21 post-transcriptional regulation, offer functional significance for the existence of multiple p21 transcripts, and support a key role for GCN2 in regulating the cell cycle under stress.

## Author Summary

Cells sense nutrient levels in their environment in order to determine if conditions are favorable to divide. GCN2 is a protein that senses amino acids and responds to amino acid deficiency by suppressing protein synthesis and increasing the expression of genes involved in recovery from nutrient stress. Although GCN2’s role in amino acid sensing is well-characterized, it is not known how it links nutrient availability with the cell cycle. Here, we show that GCN2 induces the cell cycle inhibitor p21^Cip1^ at the level of protein translation. The induction of p21 is limited to a specific messenger RNA variant that contains upstream open reading frames, and these upstream open reading frames are required for its enhanced translation under stress. Previously, the functional significance of these different p21 variants was unknown. Upregulation of p21 allows cells to halt division and survive under conditions of nutrient stress. Collectively, this work demonstrates a new mechanism of p21 regulation and the connection between GCN2 and the cell cycle.

## Introduction

p21^Cip1^, a member of the CIP/KIP family of cell cycle inhibitors, is known to play a key role in regulating the transition from G_1_ to S phase of the cell cycle. Under normal conditions, complexes of cyclin E and cyclin dependent kinase 2 (CDK2) are active in late G_1_ phase and phosphorylate the retinoblastoma protein (pRb). Phosphorylated pRB dissociates from E2F transcription factors, resulting in the transactivation of genes required for progression into S phase. Many different stress conditions upregulate p21 expression to inhibit cell proliferation and allow time for recovery before cell division. When p21 is induced, it binds to and inhibits cyclin E:CDK2 complexes. This prevents full phosphorylation of pRB, resulting in G_1_/S arrest [1].

p21 expression must be tightly controlled in order for cells to properly progress through the cell cycle. As a result, p21 is regulated at many levels, both transcriptionally and post-transcriptionally [2]. At the transcriptional level, p21 was first described as a p53 target and a major effector of cell cycle arrest in response to DNA damage [3,4]. However, many stressors, such as oncogenic Ras [5] and histone deacetylase inhibition [6], upregulate p21 transcription independently of p53. A variety of mechanisms regulate p21 levels post-transcriptionally. mRNA binding proteins, such as Hu-antigen D (HuD) [7] and Hu-antigen R (HuR) [8], bind to the 3’ untranslated region (UTR) of the p21 transcript to enhance its stability. Both ubiquitin-dependent and independent pathways regulate the stability of p21 protein [9]. Phosphorylation events also regulate p21 protein stability, as well as its binding partners and subcellular localization [10].

Interestingly, p21 is encoded by multiple transcript variants in both mice and humans [11,12]. Although much is known about the regulation of p21 expression as a whole, the regulation of the individual transcript variants is not understood. These transcript variants differ in their 5’ UTRs but are identical in their coding sequence and thus produce the same protein. The 5’ UTR of a transcript plays a crucial role in regulating its translation, suggesting that these variants might be differentially controlled at the translational level.

Here, we demonstrate that upstream open reading frames (uORFs) in the 5’ UTR of an individual p21 transcript variant upregulate its translation during amino acid deprivation. This stress activates the serine/threonine kinase general control non-derepressible 2 (GCN2), which phosphorylates the eukaryotic translation initiation factor eIF2α [13]. Mutation of the uORFs in the p21 transcript variant or mutation of eIF2α preventing its phosphorylation blocks translational induction. Similarly, the translation of a second p21 transcript variant that lacks uORFs is not enhanced by eIF2α phosphorylation. Unlike many other known mechanisms of p21 upregulation under stress, GCN2-dependent regulation does not require p53 or the other p53 family members, p63 and p73. p21 induction is required for optimal G_1_/S arrest and promotes cell survival under conditions of amino acid deprivation. Together, these findings uncover a novel mechanism of differential regulation of distinct p21 transcript variants at the translational level and establish GCN2 as a critical regulator of the cell cycle and cell survival.

## Results

### p21 accumulation in response to amino acid deprivation requires GCN2 and eIF2α phosphorylation

**Fig 1:**
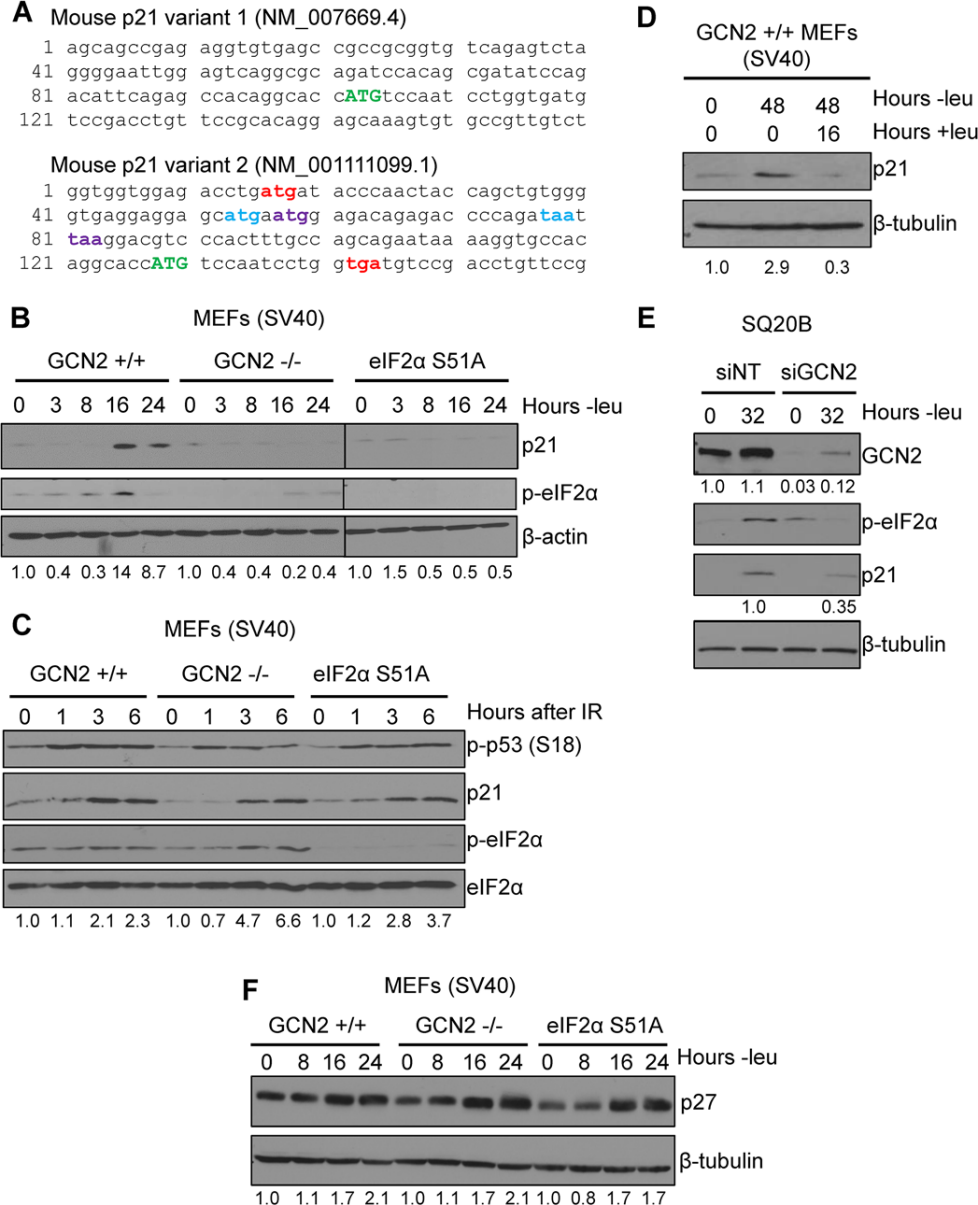
p21 induction under amino acid deprivation requires GCN2 and eIF2α phosphorylation. A) The 5’ regions of the two known mouse transcript variants of p21 are shown. The start codon for p21 is indicated in green capital letters. Variant 2 contains three 5’ uORFs; the start and stop codons for each are indicated in red, blue, and purple, respectively. B) Western blot analysis of p21 induction in leucine-deprived GCN2^+/+^, GCN2^-/-^, and eIF2α S51A MEFs. β-actin was used as a loading control. All samples were collected at the same time and the blots were run in parallel. C) Western blot analysis of p21 induction in GCN2^+/+^, GCN2^-/-^, and eIF2α S51A MEFs irradiated with a dose of 4 Gy. Total eIF2α was used as a loading control. D) Western blot analysis of the reversibility of p21 induction in GCN2^+/+^ MEFs. β-tubulin was used as a loading control. E) Western blot analysis of p21 induction in leucine-deprived SQ20B cells. Cells were transfected with either non-targeting siRNA (siNT) or siRNA against GCN2 (siGCN2). β-tubulin was used as a loading control. F) Western blot analysis of p27 induction in leucine-deprived GCN2^+/+^, GCN2^-/-^, and eIF2α S51A MEFs. β-tubulin was used as a loading control. Data information: Values below blot represent the fold change in total pixel intensity over control of p21, GCN2, or p27 normalized to the loading control for each lane.

Multiple p21 transcript variants have been identified in mammals, but their significance remains unknown. In mice, there are two p21 transcripts generated by alternative promoter usage. The classical p21 transcript is designated as variant 1, while variant 2 is produced from a promoter located 2.8 kilobases upstream from the variant 1 promoter [11]. Both transcripts are ubiquitously expressed throughout mouse tissues [11]. These variants differ only in their 5’ UTRs, suggesting that they undergo differential post-transcriptional regulation. Elements in the 5’ UTR, such uORFs and internal ribosome entry sites (IRESes), play a major role in regulating translation. Analysis of the known mouse p21 transcript variants revealed that transcript variant 2 contains 5’ uORFs, while transcript variant 1 does not. A diagram of the 5’ uORFs is depicted in Fig 1A. The first uORF of the transcript overlaps out of frame with the coding region for p21. uORFs 2 and 3 are located within the first uORF and are fully contained within the 5’ UTR. Human p21 is also encoded by multiple transcripts, some of which contain uORFs in their 5’ UTRs [12].

One of the most well-studied transcripts translationally regulated by uORFs is activating transcription factor 4 *(ATF4)*. ATF4 is a transcription factor that is translationally upregulated as part of the Integrated Stress Response (ISR). The ISR consists of the four mammalian kinases GCN2, protein kinase RNA-activated (PKR), PKR-like endoplasmic reticulum kinase (PERK) and heme-regulated inhibitor (HRI), which all phosphorylate eIF2α in response to a diverse array of stressors [14]. While eIF2α phosphorylation leads to a general downregulation of protein synthesis, the presence of 5’ uORFs in *ATF4* leads to its translational upregulation under these conditions [15,16]. Mouse *ATF4* contains two 5’ uORFs. Under normal conditions, translation begins at the first uORF and then quickly reinitiates at the second uORF. Since the second uORF overlaps out of frame with the ATF4 coding region, no protein is produced. Upon eIF2α phosphorylation, the exchange of GDP to GTP on eIF2 is hampered. This delays translation reinitiation, allowing ribosomes to scan through uORF2 and begin translation at the ATF4 ORF [15].

To determine if p21 is regulated by eIF2α phosphorylation, we cultured SV40 large T antigen transformed MEFs in media lacking leucine to activate GCN2, which phosphorylates eIF2α upon amino acid deprivation. We chose to focus on GCN2 because previous work has demonstrated that amino acid deprivation upregulates p21 [17,18]. Leucine deprivation resulted in phosphorylation of eIF2α and induction of p21. This response was strictly dependent on GCN2, as GCN2^-/-^ MEFs did not phosphorylate eIF2α or induce p21 under stress (Fig 1B). MEFs containing a knock-in mutation of eIF2α in which serine 51 is converted alanine to prevent phosphorylation were also unable to upregulate p21 under stress, indicating that GCN2-dependent phosphorylation of eIF2α is necessary for p21 induction upon amino acid starvation (Fig 1B). A close analysis of the kinetics of induction showed that p21 protein induction began between 8 and 12 hours of leucine deprivation (S1A Fig). We repeated the experiment with glutamine deprivation to ensure that these results were not specific to leucine deprivation. Similar results were observed in the three cell lines (S1B Fig).

Next, we wanted to determine if dependence on GCN2 and p-eIF2α for p21 upregulation is a general stress response phenomenon or is restricted to nutrient deprivation. To do so, we exposed cells to ionizing radiation, a stress that induces p21 but does not activate the ISR. As shown in Fig 1C, wildtype, GCN2^-/-^, and eIF2α S51A cells all rapidly increased p21 expression in response to DNA damage. These data indicate that GCN2 loss specifically renders cells incapable of inducing p21 in response to amino acid deprivation.

After resolution of stress, we found that p21 levels return to baseline. GCN2^+/+^ MEFs were cultured without leucine for two days, then returned to complete media for 16 hours. p21 induction was robust after 48 hours of amino acid deprivation, but levels were barely detectable after a period of recovery (Fig 1D). This indicates that p21 induction is reversible and cells can adjust p21 levels in response to their environment.

To confirm that these findings were not specific to MEFs, we transfected SQ20Bs, a human head and neck squamous cell carcinoma cell line, with non-targeting siRNA or siRNA against GCN2 and cultured the cells without leucine. Upon leucine deprivation, the siNT-transfected SQ20Bs phosphorylated eIF2α and induced p21. Knockdown of GCN2 prevented eIF2α phosphorylation and reduced p21 induction by almost 70% (Fig 1E). Thus, GCN2 regulates p21 in both human and murine cells.

It has been reported that amino acid deprivation also induces p27^Kip1^, a cell cycle inhibitor closely related to p21 [17]. To establish if GCN2 and eIF2α phosphorylation are responsible for its upregulation under amino acid deprivation, we leucine deprived GCN2^+/+^, GCN2^-/-^, and eIF2α S51A MEFs cells and found that all three cell lines induced p27 (Fig 1F). The p27 5’ UTR does not contain any uORFs (S1C Fig), further supporting that eIF2α is not responsible for its upregulation under stress. Thus, p21 and p27 are regulated by different mechanisms under nutrient stress.

### Transcriptional upregulation of p21 through p53 does not play a role in its induction by amino acid deprivation

**Fig 2:**
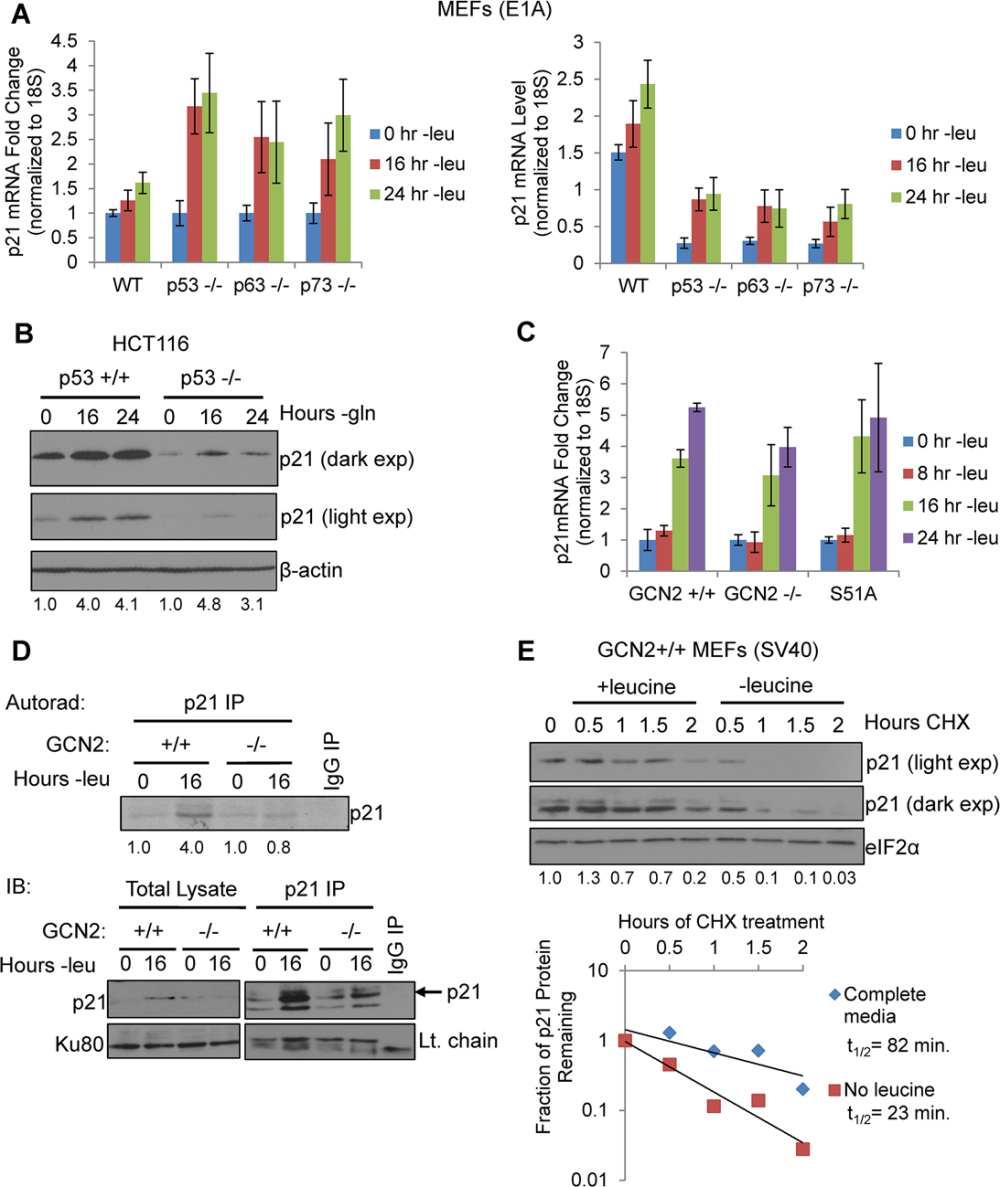
GCN2 enhances p21 translation independently of p53. A) qPCR for p21 was performed on RNA isolated from wildtype, p53^-/-^, p63^-/-^, and p73^-/-^ MEFs deprived of leucine for the indicated times. p21 transcript levels were normalized to 18S rRNA. Left: Results are depicted as fold change over control for each cell line. Right: Results are depicted as absolute levels of normalized transcript. Data represent the average of three independent experiments ± S.E.M. B) Western blot analysis of p21 induction in p53^+/+^ and p53^-/-^ HCT116s. β-actin was used as a loading control. Values below blot represent fold change in total pixel intensity over control of p21 normalized to the loading control for each lane. C) qPCR for p21 was performed on RNA isolated from GCN2^+/+^, GCN2^-/-^, and eIF2α S51A MEFs deprived of leucine for the indicated times. p21 transcript levels were normalized to 18S rRNA and are depicted as fold change over control. Data are the average of three independent experiments ± S.E.M. D) Metabolic labeling of p21 under leucine deprivation. Top: Autoradiograph of ^35^S-labeled p21 immunoprecipitated from GCN2^+/+^ and GCN2^-/-^ MEFs grown with or without leucine for 16 hours. Bottom: Western blot with cold amino acids to determine immunoprecipitation efficiency and verify induction of p21 in total cell lysates. E) Measurement of p21 protein half-life under replete and leucine starved conditions. GCN2^+/+^ MEFs were initially grown in leucine-free media to induce p21 protein levels at time 0. Cells were then switched to complete or leucine-free media containing 50 μg/mL cycloheximide. Top: Western blot analysis of p21 protein levels during a time course of cycloheximide treatment. Total eIF2α was used as a loading control. Values below blot represent fold change in total pixel intensity over control of p21 normalized to the loading control for each lane. Bottom: Normalized p21 protein values were fit to exponential decay curves to calculate protein half-life.

One of the best-known mechanisms of p21 regulation is at the transcriptional level through p53, and it has been previously established that p53 regulates both murine p21 transcript variants [11]. To evaluate if any p53 family members contribute to p21 upregulation under amino acid deprivation, we leucine starved E1A transformed wildtype, p53^-/-^, p63^-/-^, and p73^-/-^ MEFs and measured p21 transcript levels by qPCR. p21 transcript levels were upregulated in all cell lines in a time-dependent manner, although loss of any one of the p53 family members compromised basal levels of p21 mRNA (Fig 2A). To confirm these results in a human cell line, we glutamine starved p53^+/+^ and p53^-/-^ HCT116s, a human colorectal adenocarcinoma cell line, and analyzed p21 induction by western blot. Similar to the MEFs, we observed a decrease in basal levels of p21 in p53^-/-^ cells, but loss of p53 did not prevent p21 upregulation under stress (Fig 2B). Furthermore, the SQ20B cells shown in Fig 1E express a nonfunctional mutant version of p53 [19]. Overall these results demonstrate that p53 and other p53 family members maintain p21 transcript levels in unstressed cells but are not necessary for p21 induction upon amino acid deprivation.

### A p21 transcript variant containing 5’ uORFs is translationally upregulated by GCN2

When p21 mRNA levels were measured in leucine-starved GCN2^+/+^, GCN2^-/-^, and eIF2α S51A MEFs, we observed a time-dependent increase in total p21 transcript in all three cell lines (Fig 2C). Loss of GCN2 or the eIF2α S51A mutation did not affect basal levels of p21 transcript (S2A Fig). We also measured levels of the p21 transcript variants separately under leucine deprivation. Transcript variant 1 was the predominant variant present in the MEFs, with a relative abundance of six to eight times more than variant 2 in complete media (S2B Fig). Under amino acid deprivation, increases in variant 1 are primarily responsible for the increase in total p21 mRNA (S2C Fig). Interestingly, variant 2 levels increased modestly in GCN2^+/+^ and eIF2α S51A MEFs under stress, but decreased by half in GCN2^-/-^ MEFs (S2D Fig). This indicates that GCN2 plays a role independently of eIF2α in maintaining variant 2 mRNA levels under conditions of leucine deprivation.

The increase in p21 mRNA without a corresponding increase in p21 protein in the GCN2^-/-^ and eIF2α S51A MEFs further supported that p21 is regulated at the translational level by eIF2α phosphorylation through GCN2. To directly measure p21 translation, we grew GCN2^+/+^ and GCN2^-/-^ MEFs with or without leucine for 16 hours, labeled newly synthesized proteins with ^35^S-methionine and ^35^S-cystiene, and immunoprecipitated p21. GCN2^+/+^ MEFs increased translation of p21 by approximately four-fold under leucine deprivation, while GCN2^-/-^ MEFs showed no increase in p21 labeling under stress (Fig 2D). Thus, GCN2 translationally upregulates p21 under nutrient stress.

We next wanted to address the possibility that enhanced protein stability might also contribute to increased p21 levels under amino acid deprivation. We leucine starved GCN2^+/+^ MEFs to initially induce p21 protein, and then switched the cells to complete or leucine-free media containing cycloheximide to block any new p21 protein synthesis. A time course analysis revealed that p21 protein half-life was approximately 80 minutes in complete media and 20 minutes in leucine-free media (Fig 2E). Therefore, enhanced protein stability does not contribute to p21 induction under nutrient stress. Furthermore, this experiment further supports the notion that translational control plays a major role in maintaining p21 protein levels under stress.

**Fig 3:**
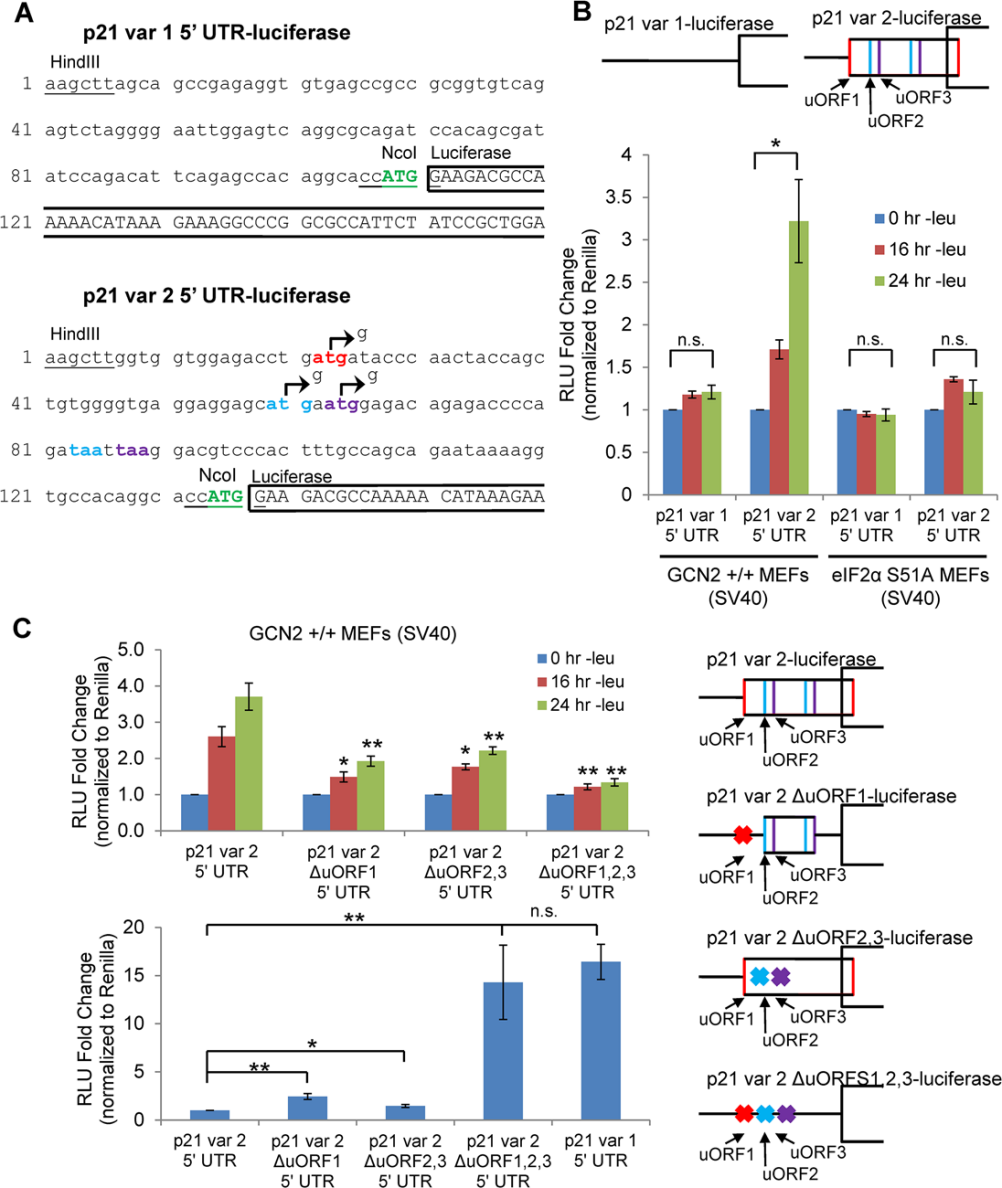
GCN2-dependent translational upregulation of p21 is specific to variant 2 and mediated by the presence of 5’ uORFs. A) Partial sequence of p21 5’ UTR luciferase reporter constructs. The p21 sequence is in lowercase letters, and the luciferase sequence is in capital letters. Restriction sites used to insert the p21 5’ UTRs are underlined. The start codon for p21 is indicated in green capital letters. The start and stop codons for p21 variant 2 uORFs 1, 2, and 3 are in red, blue, and purple letters, respectively. The mutations created to disrupt the uORFs are indicated by arrows. B) Luciferase assay using p21 5’ UTR reporter constructs under leucine deprivation. Top: A schematic representation of the p21 variant 1 and 2 luciferase reporter constructs (not drawn to scale). Bottom: Luciferase activity from reporter constructs was measured in leucine-deprived GCN2^+/+^ and eIF2α S51A MEFs and normalized to *Renilla* luciferase. Data are the average of three independent experiments ± S.E.M.; *p<0.05. C) Luciferase assay using mutant p21 variant 2 5’ UTR reporter constructs. Right: A schematic representation of the p21 variant 2 luciferase reporter constructs (not drawn to scale). Top left: Luciferase activity from reporter constructs was measured in leucine-deprived GCN2^+/+^ MEFs. Bottom left: Relative basal translation levels of mutant p21 variant 2 reporter constructs in untreated cells. All results were normalized to *Renilla* luciferase. Data are the average of three (p21 variant 1 and p21 variant 2 ∆uORF1,2,3) or four (remaining constructs) independent experiments ± S.E.M.; *p<0.5, **p<0.01.

We created luciferase reporter constructs to assess the effects of amino acid deprivation on the translational efficiency of each p21 variant. To do so, we inserted either the 5’ UTR of p21 variant 1 or 2 between the HindIII and NcoI restriction sites in a pGL3 reporter vector in which the SV40 promoter drives expression of luciferase. This created a construct in which the luciferase coding sequence is located directly after the p21 ORF start codon (Fig 3A). GCN2^+/+^ MEFs were transfected with the resulting p21 variant 1 5’ UTR-luciferase and p21 variant 2 5’ UTR-luciferase plasmids, and luciferase activity was measured during a time course of leucine deprivation. No change in luciferase activity was observed from the variant 1 construct, while luciferase activity from the p21 variant 2 construct increased in a time-dependent manner (Fig 3B). These results indicate that translational upregulation is specific to p21 variant 2. Furthermore, the translational regulation was dependent on eIF2α phosphorylation, as there was no statistically significant increase in luciferase activity from the variant 2 construct in eIF2α S51A MEFs (Fig 3B).

In order to determine if the 5’ uORFs in the p21 variant 2 transcript were responsible for translational upregulation under stress, we introduced point mutations to disrupt the uORF start codons. Constructs were created with deletion of uORF1 (ΔuORF1), deletion of both uORFs 2 and 3 (ΔuORF2,3), and deletion of all three uORFs (ΔuORF1,2,3) (Fig 3A). Again, GCN2^+/+^ MEFs were transfected with these constructs, and luciferase activity was measured over a time course of leucine deprivation. Deletion of uORF1 or deletion of both uORFs 2 and 3 compromised induction of luciferase activity, demonstrating that all three uORFs promote translation under stress. The uORFs appear to be the only 5’ elements regulating p21 translation since deletion of all three uORFs essentially blocked the increase in luciferase activity (Fig 3C). Under basal conditions, deletion of either uORF1 or uORFs 2 and 3 modestly raised luciferase activity, indicating that all three uORFs are inhibitory to translation under normal, unstressed conditions. The deletion of all three uORFs completely rescued basal luciferase activity to levels similar to that of p21 variant 1, which contains no uORFs (Fig 3C). This suggests that under unstressed conditions, ribosomes tend to initiate translation at the uORFs instead of the p21 ORF.

To confirm that the changes observed in luciferase activity were not due to changes in the transcript levels of the reporters, we performed qPCR for luciferase on GCN2^+/+^ MEFs transfected with the 5’ UTR constructs. No appreciable changes in luciferase mRNA were observed (S3A Fig). As a positive control, luciferase assays were performed with ATF4 5’ UTR constructs (S3B Fig). In agreement with previous data [15], treatment with thapsigargin induced luciferase activity from the wildtype construct, and deletion of uORF2 increased basal translation levels while preventing induction in response to stress. This response is very similar to that seen with the deletion of p21 variant 2 uORFs.

**Fig 4:**
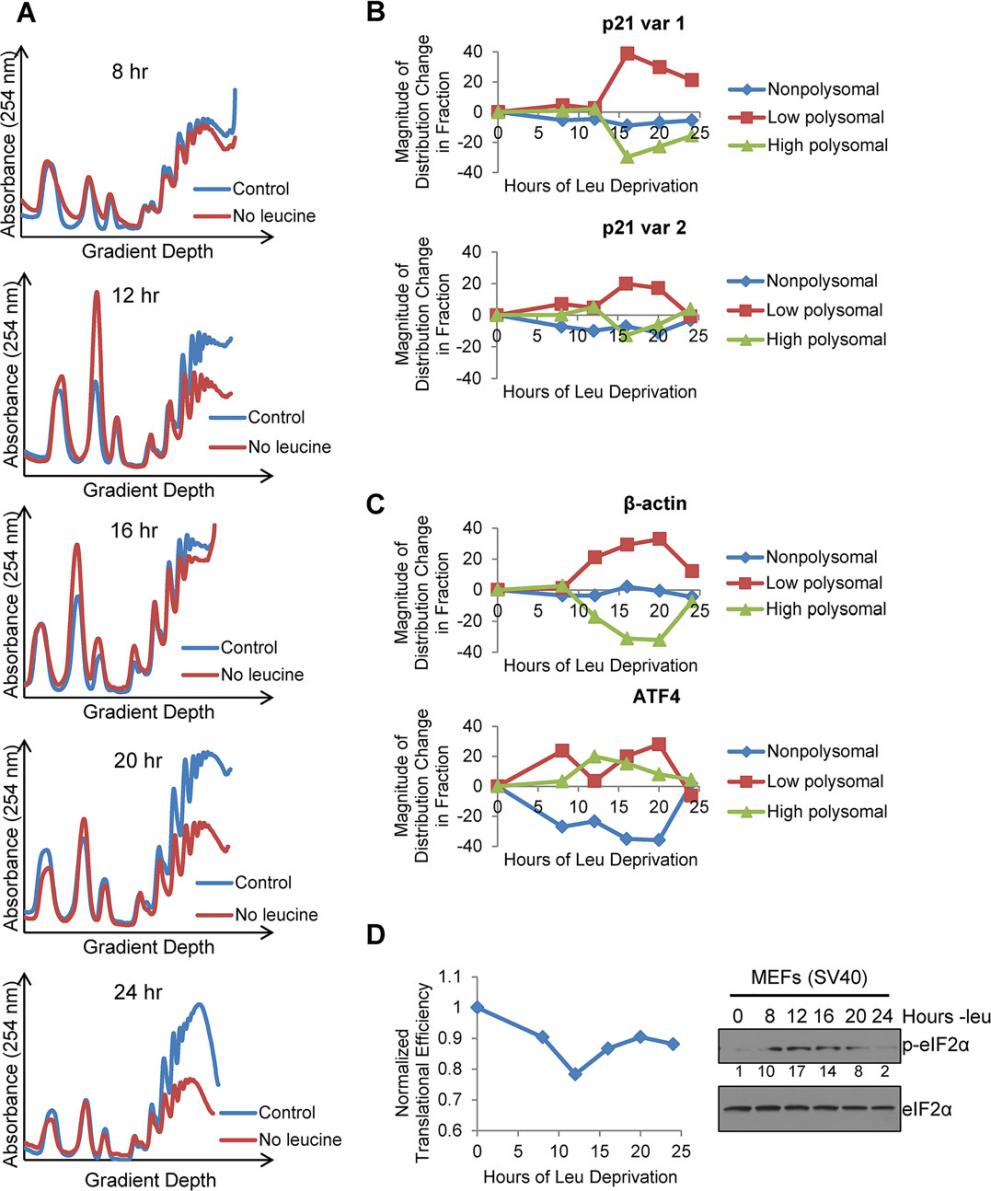
Polysome profiling reveals differences between the translational efficiencies of p21 variants 1 and 2 under nutrient stress. A)Polysome profiles of leucine-starved GCN2^+/+^ MEFs over time. Lysates from GCN2^+/+^ MEFs grown with or without leucine for 8, 12, 16, 20, and 24 hours were separated on 10 to 50% sucrose gradients. Polysome profiles of the gradients were generated by measuring absorbance at 254 nm. B) Time course of p21 translational efficiency as measured by mRNA association with polysomes. qPCR was performed on fractions pooled from sucrose gradients as indicated in S4A Fig p21 transcript levels in each group were normalized to total transcript. C) Time course of β-actin and ATF4 translational efficiency as measured by mRNA association with polysomes. qPCR was performed on fractions pooled from sucrose gradients as indicated in S4A Fig. Transcript levels in each group were normalized to total transcript. D) Time course of global translational efficiency in leucine-deprived GCN2^+/+^ MEFs. Left: Time course of translational efficiency in leucine-deprived cells. Right: Western blot analysis of p-eIF2α in leucine-deprived GCN2^+/+^ MEFs. Values below blot represent the fold change in total pixel intensity of p-eIF2α over control normalized to eIF2α.

To ascertain if the two endogenous variants of p21 mRNA were translated with different efficiencies under stress, we leucine starved GCN2^+/+^ MEFs for 8, 12, 16, 20, and 24 hours, and analyzed the distribution of the two p21 transcript variants in different polysome fractions. The polysome profiles for these time points are shown in Fig 4A, and the pooling scheme of the fractions for qPCR analysis is shown in S4A Fig p21 variant 1 exhibited a strong shift from the high to the low molecular weight polysomes at 16 hours, after which it began to recover towards its baseline distribution (Fig 4B). This pattern of translational suppression and recovery is similar to that of the housekeeping gene β-actin, as opposed to ATF4, which is translationally upregulated under stress (Fig 4C). The association of p21 variant 2 with polysomes changed very little with time. It exhibited a slight decrease in association at 16 hours, followed by full recovery back to baseline at 24 hours (Fig 4B). Thus, by 24 hours, p21 variant 2 appears to be translated at its normal efficiency while variant 1 is still suppressed (Fig 4B and S4B Fig).

To determine the effect of leucine deprivation on global translation, we used the polysome profiles shown in Fig 4A to calculate translational efficiency at each time point as previously described [20] and normalized to the translational efficiency in complete media. This value reflects the fraction of all mRNAs that are associated with polysomes under leucine deprivation as compared to complete media. Global translational efficiency reached its nadir of approximately 0.75 at 12 hours of leucine deprivation and then recovered to a nearly constant level of 0.9 (Fig 4D). The kinetics of translational efficiency closely correlated with eIF2α phosphorylation, which reached its peak at 12 hours of leucine deprivation and then decreased to near baseline levels by 24 hours (Fig 4D). Additionally, we observed decreased levels of eIF4G and hypophosphorylation of 4E-BP1, which may also contribute to translational suppression (S4C Fig). Leucine deprivation also increased phosphorylation of the elongation factor eEF2 (S4C Fig). However, inhibition of elongation by eEF2 phosphorylation would not alter mRNA association with polysomes [21].

### p21 contributes to cell cycle arrest and promotes cell survival under conditions of amino acid deprivation

**Fig 5:**
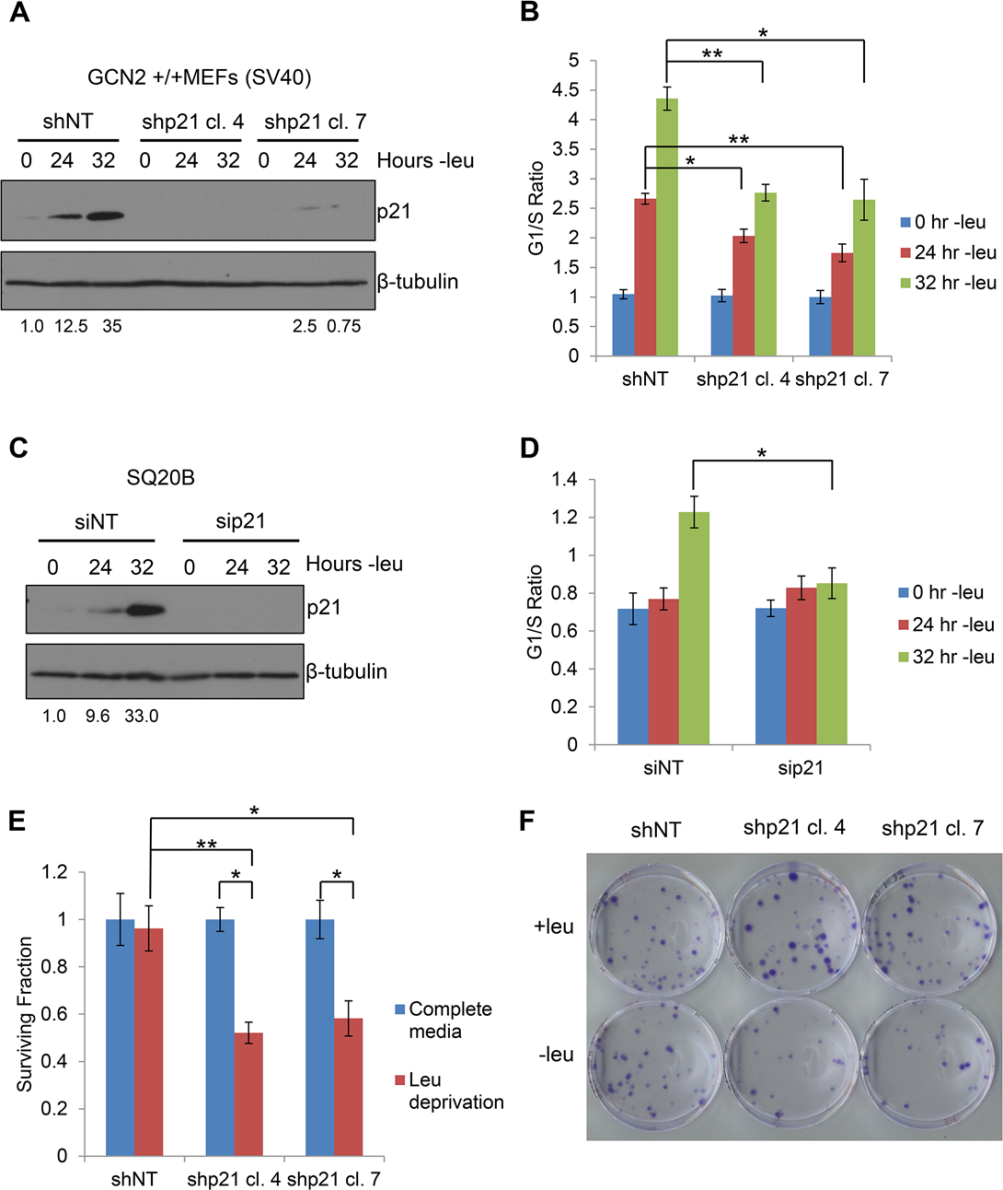
p21 regulates G_1_/S arrest and cell survival under conditions of amino acid deprivation. A) Western blot analysis of the MEFs used in (B) to determine p21 knockdown efficiency. Values below blot represent the fold change in total pixel intensity over control of p21 normalized to the loading control for each lane. β-tubulin was used as a loading control. B) G_**1**_/S ratio of leucine-starved GCN2^+/+^ MEFs stably transfected with non-targeting shRNA (shNT) or shRNA against p21 (shp21). DNA content was measured by propidium iodide staining and flow cytometry analysis. Data are the average of three independent experiments ± S.E.M.; * p<0.05, ** p<0.01. C) Western blot analysis of the SQ20Bs used in (D) to determine p21 knockdown efficiency. Values below blot represent the fold change in total pixel intensity over control of p21 normalized to the loading control for each lane. β-tubulin was used as a loading control. D) G_**1**_/S ratio of leucine-starved SQ20Bs transfected with non-targeting siRNA (siNT) or siRNA against p21 (sip21). DNA content was measured by propidium iodide staining and flow cytometry analysis. Data are the average of four (siNT) or five (sip21) independent experiments ± S.E.M.; * p<0.05, ** p<0.01. E) Clonogenic survival of control and p21 knockdown GCN2^+/+^ MEFs exposed to long-term leucine starvation. Data are the average of three independent experiments ± S.E.M; *p< 0.5, **p< 0.1. F) Representative picture of control and p21 knockdown MEFs from the clonogenic survival assay described in (E) after one week of colony formation.

Next, we sought to determine the functional consequences of p21 upregulation under amino acid starvation. We hypothesized that wildtype cells would undergo G_1_/S arrest in response to amino acid deprivation, while cells lacking p21 would continue to proliferate. To this end, we stably transfected GCN2^+/+^ MEFs with either non-targeting shRNA (shNT) or shRNA directed against p21 (shp21). The knockdown efficiency of two independent shp21 clones is shown in Fig 5A. Leucine starvation resulted in a time-dependent increase in the G_1_/S ratio of GCN2^+/+^ shNT MEFs (Fig 5B), as measured by flow cytometry. Knockdown of p21 resulted in a statistically significant decrease in the G_1_/S ratio in both clones, indicating that p21 promotes cell cycle arrest under amino acid deprivation. The gating strategy used to select single cells for cell cycle analysis is shown in S5A Fig.

To confirm these results in a human cell line, we transfected SQ20B cells with either non-targeting siRNA (siNT) or siRNA against p21 (sip21). The knockdown efficiency of p21 is shown in Fig 5C. An increase in the G_1_/S ratio was observed in siNT transfected cells at 32 hours of leucine deprivation, while no appreciable change in the G_1_/S ratio was detected in sip21 transfected cells (Fig 5D). Thus, p21 regulates G_1_/S arrest under amino acid deprivation in both murine and human cells.

To directly assess the role of GCN2 and eIF2α phosphorylation in cell cycle arrest, we leucine starved GCN2^-/-^ and eIF2α S51A MEFs and measured the degree of G_1_/S cell cycle arrest by flow cytometry. The phenotype was even stronger in these cells, as they did not undergo any measurable degree of cell cycle arrest by 32 hours of leucine starvation (S5B Fig and S5C Fig). This indicates that GCN2 and eIF2α phosphorylation are critically required for the induction of G_1_/S arrest in response to leucine deprivation and likely regulate other factors that promote cell cycle arrest under stress. These results were also confirmed in SQ20B cells transfected with siNT or siGCN2 (S5D Fig).

Previous findings showed that ER stress-induced phosphorylation of eIF2α by PERK and GCN2 in NIH 3T3 cells resulted in translational suppression of cyclin D1 and subsequent G_1_ arrest [22,23]. To determine if a similar scenario occurred in our system, we measured levels of cyclin D1 protein in leucine-starved GCN2^+/+^ and GCN2^-/-^ MEFs. S5E Fig shows that cyclin D1 levels decreased over time in both cell lines. Therefore, under our study conditions, GCN2 does not contribute to cell cycle regulation by modulating levels of cyclin D1. The GCN2-independent decrease in cyclin D1 and increase in p27 may explain why knockdown of p21 does not completely abolish the ability of MEFs to undergo G_1_/S arrest in response to amino acid deprivation.

Next, we wanted to determine if p21 contributes to cell survival under amino acid deprivation. To do so, we cultured the shNT and shp21 MEF clones in complete media or media lacking leucine. After 72 hours, the viable cells were re-plated at low density in complete media. Virtually all shNT cells were able to recover from nutrient stress and form colonies. However, clonogenic survival was significantly reduced by approximately 40–50% in the two shp21 MEF clones after leucine deprivation (Fig 5E–5F). Importantly, knockdown of p21 did not compromise cell viability, as measured by trypan blue exclusion, or clonogenic survival (S6A–S6B Fig) when the cells were grown in complete media. These results demonstrate that p21 is dispensable for cell growth under nutrient replete conditions but is critical for cell survival during the recovery from amino acid starvation. Interestingly, these results are consistent with another study which showed that p21 promotes cell survival under the combined stresses of complete amino acid and serum starvation independently of p53 [24].

## Discussion

Cells have evolved multiple mechanisms to regulate p21 at the post-transcriptional level. However, translational regulation of p21 has been largely unexplored. Here, we demonstrate a novel mechanism of translational control of a specific p21 transcript variant that regulates cell proliferation and survival under nutrient stress. GCN2 activation and subsequent phosphorylation of eIF2α translationally upregulates a p21 mRNA variant with 5’ uORFs. Many genes are upregulated by eIF2α phosphorylation indirectly through activation of the ATF4 transcriptional program [25,26]. However, the number of genes known to be directly translationally upregulated by eIF2α phosphorylation is much smaller. In addition to ATF4, several other genes, such as activating transcription factor 5 (ATF5) [27], CCAAT/enhancer-binding protein homologous protein (CHOP) [28], growth arrest and DNA-damage-inducible 34 (GADD34) [29], inhibitor of Bruton tyrosine kinase α (IBTKα) [30], and protein kinase C η (PKCη) [31], are also translationally upregulated by eIF2α phosphorylation due to the presence of uORFs in the 5’ UTR. These studies into p21 regulation expand on the known targets of translational upregulation by phosphorylation of eIF2α and provide greater understanding of the functional outcomes of ISR activation.

**Fig 6:**
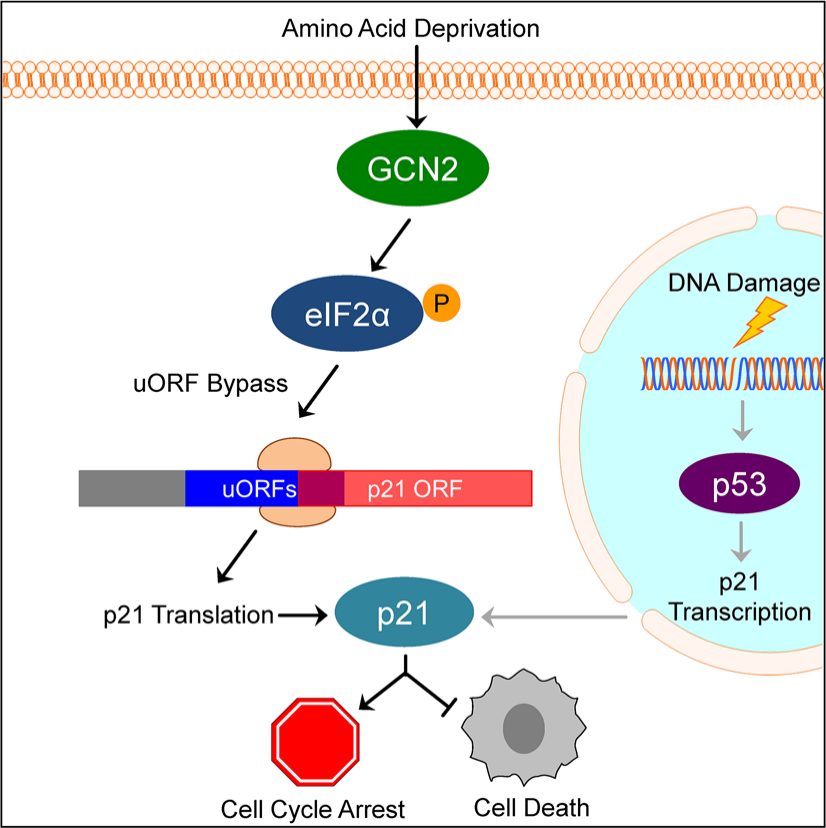
Model of p21 translational regulation. Upon amino acid deprivation, GCN2 phosphorylates eIF2α, which delays translation initiation. This results in ribosomes bypassing 5’ uORFs in the p21 variant 2 transcript and beginning translation at the p21 ORF, resulting in p21 upregulation under stress. This pathway is distinct from p53-dependent transcriptional upregulation of p21. Induction of p21 upon nutrient deprivation leads to G_**1**_/S arrest and promotes cell survival.

Our results suggest a model in which under normal, unstressed conditions, translation typically initiates at the uORFs in the p21 variant 2 transcript, resulting in very little production of p21 protein. Initiation at uORF1 would fail to produce a protein because it overlaps out of frame with the p21 coding sequence. Initiation at uORFs 2 and 3 likely do not produce a protein because they are located very close to the p21 coding sequence, which does not provide ribosomes sufficient time to reinitiate translation at the p21 ORF. When GCN2 phosphorylates eIF2α, translation initiation is delayed. This promotes leaky scanning through the uORFs and increases the likelihood that ribosomes will begin translation at the p21 ORF (Fig 6).

This mechanism of translation regulation also offers an explanation for the existence of multiple p21 transcript variants that encode for the same protein. While our work focused on the two mouse p21 mRNA variants, humans also have multiple p21 mRNA variants that are likely regulated in a similar manner [12]. Some of these variants contain 5’ uORFs, while others contain none. We speculate that p21 transcript variants with 5’ uORFs provide an advantage to cells by allowing them to upregulate p21 under stress conditions that would normally repress translation. Translation is one of the highest energy consuming processes in the cell, which is why it is often suppressed to conserve resources under stress. Under these conditions, proliferation is not favored and upregulation of p21 provides an advantage to cells by promoting cell cycle arrest. However, the variants without 5’ uORFs ensure that cells are not restricted to express p21 only when eIF2α is phosphorylated. Other stresses, such as DNA damage, do not suppress translation but still require cell cycle arrest to allow time for recovery. In these cases, cells could still translate p21 efficiently to respond to stress even though eIF2α remains unphosphorylated.

From a functional standpoint, this study provides a link between the nutrient-sensing capabilities of GCN2 and the mammalian cell cycle. Several studies have shown that Gcn2 regulates a G_1_ checkpoint in the fission yeast *Schizosaccharomyces pombe* in response to stressors such as nitrogen starvation [32] and UV irradiation [33], but GCN2’s contribution to this process in mammalian cells was not delineated. Many mammalian proteins that respond to nutrient levels impinge upon the cell cycle. Amino acids are also sensed by mTOR, one of the master metabolic switches of the cell. mTOR activity is inhibited upon amino acid deprivation, resulting in G_1_ arrest through decreased production and increased turnover of cyclin D1 [34,35] and upregulation of the cell cycle inhibitor p27 [36]. Amino acid starvation also increases the half-life of both p21 and p27 mRNA [17]. Glucose deprivation activates AMPK, a major cellular energy regulator, which phosphorylates and stabilizes p53, resulting in p21 induction [37]. Glucose deprivation also perturbs protein folding and activates the ISR initiator and endoplasmic reticulum resident kinase PERK, which induces G_1_ arrest by suppressing cyclin D1 translation [22]. Here, we demonstrate that GCN2 directly links amino acid availability with cell cycle progression by translationally upregulating a transcript variant of p21. As there is no homologue of p21 in fission yeast, cell cycle arrest downstream of Gcn2 in *S*. *pombe* must occur through a different mechanism. Based on this work, it is tempting to speculate that the *S*. *pombe* CDK inhibitor Rum1 contributes to this response, especially since there are two transcript variants of *rum1*, one that contains 5’ uORFs and one that does not [38].

The induction of p21 promotes cell cycle arrest when nutrients are scarce and conditions are not favorable for division. As a likely consequence of continued proliferation in a nutrient-poor environment, cells lacking p21 exhibit decreased survival after exposure to amino acid deprivation. These findings may have important implications for cancer. Tumor cells often exist in a nutrient-poor microenvironment due to inadequate tumor perfusion and increased metabolic demands of tumor cells [39]. We have previously shown that GCN2 is overexpressed in human tumors, and loss of GCN2 slows or inhibits tumor growth in mouse xenograft models of cancer [18]. Downstream of GCN2, p21 may serve to promote tumor cell survival in areas where nutrients are limiting. This fits with observations that p21 has both pro- and anti-tumorigenic properties [40]. In general, limiting cell division would slow tumor growth. However, in areas lacking adequate nutrient supply, p21-dependent cell cycle arrest would allow tumor cells to remain dormant and survive adverse conditions that would otherwise lead to cell death. Understanding the mechanism of p21 regulation and its function will greatly enhance the development of targeted therapeutics against the GCN2-eIF2α-p21 pathway.

## Materials and Methods

### Cell culture, transfection, and plasmids

All MEF cell lines and SQ20B cells were maintained in Dulbecco’s Modified Eagle Medium (DMEM) supplemented with 10% fetal bovine serum (FBS), penicillin, and streptomycin. GCN2^-/-^ and eIF2α S51A MEFs were additionally grown with nonessential amino acids and 55 μM β-mercaptoethanol. HCT116 cell lines were maintained in McCoy’s 5A media supplemented with 10% FBS, penicillin, and streptomycin. GCN2^+/+^ MEFs were stably transfected with pSM2-shNT (Open Biosystems) or pLKO-shp21 (Sigma) plasmids using Lipofectamine 2000 (Invitrogen) according to the manufacturer’s protocol. SQ20B cells were transfected with ON-TARGETplus SMARTpool siNT, siGCN2, or sip21 (Thermo Scientific) using Lipofectamine RNAiMAX (Invitrogen) according to the manufacturer’s protocol.

### Western blotting

Cells were lysed in Nonidet P-40 (NP-40) buffer (1% NP-40, 1 mM phenylmethylsulfonyl fluoride, 1X Complete Mini protease inhibitor cocktail [Roche], 1X phosphatase inhibitor cocktail 2 [Sigma] in PBS). Protein concentrations of lysates were determined using 660 nm Protein Assay Reagent (Thermo Scientific), according to the manufacturer’s protocol. Equal amounts of protein were resolved on sodium dodecyl sulfate polyacrylamide gels and transferred to polyvinylidene fluoride membranes. Membranes were blocked with 5% non-fat dried milk in TBS-T (20 mM Tris, 137 mM NaCl, 0.1% Tween-20; pH 7.6) and then incubated in primary antibody. Following primary antibody incubation, membranes were washed three times with 1% non-fat dried milk in TBS-T, incubated in secondary antibody, and then washed three more times with 1% non-fat dried milk in TBS-T. Proteins were visualized by incubating membranes in ECL chemicals and exposing to film. Bands were quantified using ImageJ software. The following primary antibodies were used for western blotting: 4E-BP1, β-tubulin, cyclin D1, eIF2α, eIF4E, eIF4G, GCN2, Ku80, p27, p-4E-BP1, p-eEF2, p-eIF2α, p-p53 (all from Cell Signaling); β-actin (Sigma), and p21 (BD Pharmigen). The secondary antibodies used were anti-rabbit HRP and anti-mouse HRP from Thermo Scientific.

### Metabolic labeling

GCN2^+/+^ and GCN2^-/-^ MEFs were grown in complete or leucine-free media for 16 hours. To label newly synthesized proteins, cells were methionine and cysteine starved for 30 minutes and then labeled with 75 μCi/mL of Easy Tag Express Protein Labeling Mix (Perkin Elmer) for 30 minutes. Cells were washed with PBS and lysed in NET-2 lysis buffer (50 mM Tris, 75 mM NaCl, 1% NP-40, 1 mM phenylmethylsulfonyl fluoride, 1X Complete Mini protease inhibitor cocktail [Roche], 1X phosphatase inhibitor cocktail 2 [Sigma]; pH 7.4). Lysates were precleared with protein A/G agarose beads. 300 μg of lysate was incubated with 1 μg of p21 antibody or 1 μg of mouse IgG conjugated to protein A/G agarose beads overnight at 4°C with end-over-end tumbling. Beads were washed four times with NET-2 wash buffer (50 mM Tris, 75 mM NaCl, 0.05% NP-40; pH 7.4) and one time with PBS. Immunoprecipitates were resolved on sodium dodecyl sulfate polyacrylamide gels. Gels were incubated in fixing solution (10% glacial acetic acid, 20% methanol, 3% glycerol), washed in deionized water, and incubated in Enlightening Autoradiography Enhancer (Perkin Elmer). Gels were air-dried overnight and exposed to film at -80°C.

### Plasmid construction and luciferase assay

p21 5’ UTR luciferase reporter constructs were generated by inserting the 5’ UTR sequences of the two known mouse p21 mRNA variants between the HindIII and NcoI restriction sites in the pGL3-Promoter vector (Promega). The 5’ UTRs were purchased as oligonucleotides from Integrated DNA Technologies. Dual luciferase assays (Promega) were performed according to the manufacturer’s instructions. Cells were co-transfected with *Renilla* luciferase (pRL-TK vector, Promega) for normalization.

### Polysome extraction

Polysome extractions were performed as previously described [41]. Isolated RNA further underwent two rounds of lithium chloride precipitation and purification over an RNeasy column (QIAgen). Purified RNA was then subjected to qPCR analysis, as described below.

### qPCR

RNA isolated from polysome fractions or total RNA harvested from cells using TRIzol (Ambion) was subjected to qPCR analysis. RNAs were reversed transcribed using AMV reverse transcriptase (Promega) and random hexamer primers (Invitrogen). The resulting cDNAs served as templates for qPCR using Power SYBR Green PCR Master Mix (Applied Biosystems). Data were analyzed using the Applied Biosystems 7300 System Software. PCR reaction conditions were 50°C for two minutes, 95°C for 10 min, followed by 40 cycles of 95°C for 15 seconds and 60°C for one minute. Primers used are in S1 Table.

### Cell cycle analysis

Cells were harvested, washed with 1% FBS in PBS, resuspended in PBS, and fixed in ice-cold ethanol. The fixed cells were washed with 1% FBS in PBS, resuspended in PBS, and treated with phosphate citric acid buffer (192 mM Na_2_HPO_4_; 4 mM citric acid, pH 7.8). Cells were resuspended in PI/RNase buffer (50 μg/mL propidium iodide, 267 μg/mL RNase A, 1% FBS in PBS) to stain DNA. DNA content was measured using a FACSCalibur flow cytometer on populations of 20,000 cells per sample. The percentage of cells in each phase of the cell cycle was calculated using Flo Jo software.

### Clonogenic survival assay

Cells were grown in media with or without leucine. After 72 hours, viable cells were replated at low density in complete media and incubated for one week. Colonies were fixed with a solution of 10% methanol and 10% acetic acid and then stained with 0.4% crystal violet in 20% ethanol.

### Trypan blue exclusion assay

Cells were harvested, resuspended in PBS, and diluted 1:2 in 0.4% trypan blue. Viable and nonviable cells were counted using a hemocytometer. Results were normalized to viability in complete media.

### Statistical analysis

All statistical analyses were performed using a two-tailed Student’s t test assuming homoscedasticity. A p value < 0.05 was considered statistically significant.

## Supporting Information

S1 Fig p21 and p27 are differentially regulated under amino acid deprivation. A) Western blot analysis of p21 induction in leucine-deprived GCN2^+/+^ MEFs. β-tubulin was used as a loading control. Values below blot represent the fold change in total pixel intensity over control of p21 normalized to the loading control for each lane. B) Western blot analysis of p21 induction in glutamine-deprived GCN2^+/+^, GCN2^-/-^, and eIF2α S51A MEFs. β-tubulin was used as a loading control. Values below blot represent the fold change in total pixel intensity over control of p21 normalized to the loading control for each lane. C) The 5’ region of mouse p27 mRNA is shown, and the start codon is indicated in green capital letters. There is no upstream start codon present in the 5’ UTR.
(TIF)

S2 Fig GCN2 does not affect basal levels of p21 transcript variants, but does regulate variant 2 levels under amino acid deprivation. A) qPCR for p21 was performed on RNA isolated from GCN2^+/+^, GCN2^-/-^, and eIF2α S51A MEFs grown in complete media. p21 transcript levels were normalized to 18S rRNA. Data represent the average of three independent experiments ± S.E.M. Results were not statistically significant. B) qPCR for p21 variants 1 and 2 was performed on RNA isolated from GCN2^+/+^, GCN2^-/-^, and eIF2α S51A MEFs grown in complete media. Levels of transcript variant 1 relative to transcript variant 2 were calculated using the ΔΔC_t_ method using 18S rRNA as the reference gene. Data represent the average of three independent experiments ± S.E.M. Results were not statistically significant. C) qPCR for p21 variant 1 was performed on RNA isolated from GCN2^+/+^, GCN2^-/-^, and eIF2α S51A MEFs deprived of leucine for the indicated times. p21 variant 1 transcript levels were normalized to 18S rRNA. Results are depicted as fold change over control for each cell line. Data represent the average of four independent experiments ± S.E.M. There was no statistically significant difference in variant 1 induction among the three cell types. D) qPCR for p21 variant 2 was performed on RNA isolated from GCN2^+/+^, GCN2^-/-^, and eIF2α S51A MEFs deprived of leucine for the indicated times. p21 variant 2 transcript levels were normalized to 18S rRNA. Results are depicted as fold change over control for each cell line. Data represent the average of four independent experiments ± S.E.M.; *p<0.05, **p<0.01
(TIF)

S3 Fig Control experiments for 5’ UTR-luciferase reporter assays. A) qPCR for luciferase was performed on RNA isolated MEFs transfected with the p21 5’ UTR reporter constructs. Luciferase transcript levels were normalized to 18S rRNA and are depicted as fold change over control. Data represent the average of three independent experiments ± S.E.M. No changes in luciferase transcript levels were statistically significant. B) Dual luciferase assay using mutant ATF4 5’ UTR reporter constructs. Left: Luciferase activity from reporter constructs was measured in GCN2^+/+^ MEFs treated with 1 μM thapsigargin for 6 hours and normalized to *Renilla* luciferase activity. Right: Relative basal translation levels of ATF4 reporter constructs in untreated cells as measured by luciferase assay. Results are normalized to *Renilla* and depicted as fold change over the wildtype construct. Data represent the average of three independent experiments ± S.E.M.
(TIF)

S4 Fig Translation regulation under leucine deprivation. A) Scheme of pooling sucrose gradient fractions into nonpolysomal, low molecular weight polysomal, and high molecular weight polysomal groups for qPCR analysis. This polysome profile is the 24 hour time point from Fig 4A. B) qPCR for both variants of p21 was performed on fractions pooled from sucrose gradients as indicated in (A). p21 transcript levels in each group were normalized to total transcript. Data are the average of three independent experiments ± S.E.M.; **p<0.01. C) Western blot analysis of various components of the translational machinery in leucine-deprived GCN2^+/+^ MEFs. β-tubulin was used as a loading control. Values below each blot represent the fold change in total pixel intensity over control normalized to β-tubulin (for eIF4G, eIF4E, p-eEF2) or 4E-BP1 (for p-4E-BP1).
(TIF)

S5 Fig GCN2 and eIF2α phosphorylation regulate the cell cycle under amino acid deprivation. A) Gating strategy used to select single cells for cell cycle analysis by flow cytometry. B) G_1_/S ratio of GCN2^-/-^ MEFs starved of leucine for the indicated times. DNA content was measured by propidium iodide staining and flow cytometry analysis. Changes in the G_1_/S ratio were not statistically significant. Data represent the average of three independent experiments ± S.E.M. C) G_1_/S ratio of eIF2α S51A MEFs starved of leucine for the indicated times. DNA content was measured by propidium iodide staining and flow cytometry analysis. Changes in the G_1_/S ratio were not statistically significant. Data represent the average of three independent experiments ± S.E.M. D) G_1_/S ratio of leucine-starved SQ20Bs transfected with non-targeting siRNA (siNT) or siRNA against GCN2 (siGCN2). DNA content was measured by propidium iodide staining and flow cytometry analysis. The knockdown efficiency of siGCN2 is demonstrated in Fig 1E. Data represent the average of three independent experiments ± S.E.M.; ** p<0.01. E) Western blot analysis of cyclin D1 in leucine-starved GCN2^+/+^ and GCN2^-/-^ MEFs. Ku80 was used as a loading control.
(TIF)

S6 Fig p21 does not affect cell viability or clonogenic survival when cells are grown in complete media. A) Viability of shNT, shp21 cl. 4, and shp21 cl. 7 MEFs grown in complete media as measured by trypan blue exclusion. There was no statistically significant difference in viability between shNT and shp21 cells. Data represent the average of three independent experiments ± S.E.M. B) Clonogenic survival of shNT, shp21 cl. 4, and shp21 cl. 7 MEFs grown in complete media. There was no statistically significant difference in survival between shNT and shp21 cells. Data represent the average of three independent experiments ± S.E.M.
(TIF)

S1 Table Primer sequences used for qPCR analysis. (DOCX)
