# Supplementary figures and images for: Translational Upregulation of an Individual p21Cip1 Transcript Variant by GCN2 Regulates Cell Proliferation and Survival under Nutrient Stress
Source: PLoS Genet. 2015 Jun 23;11(6):e1005212. doi: 10.1371/journal.pgen.1005212 (PMC4477940; doi:10.1371/journal.pgen.1005212)

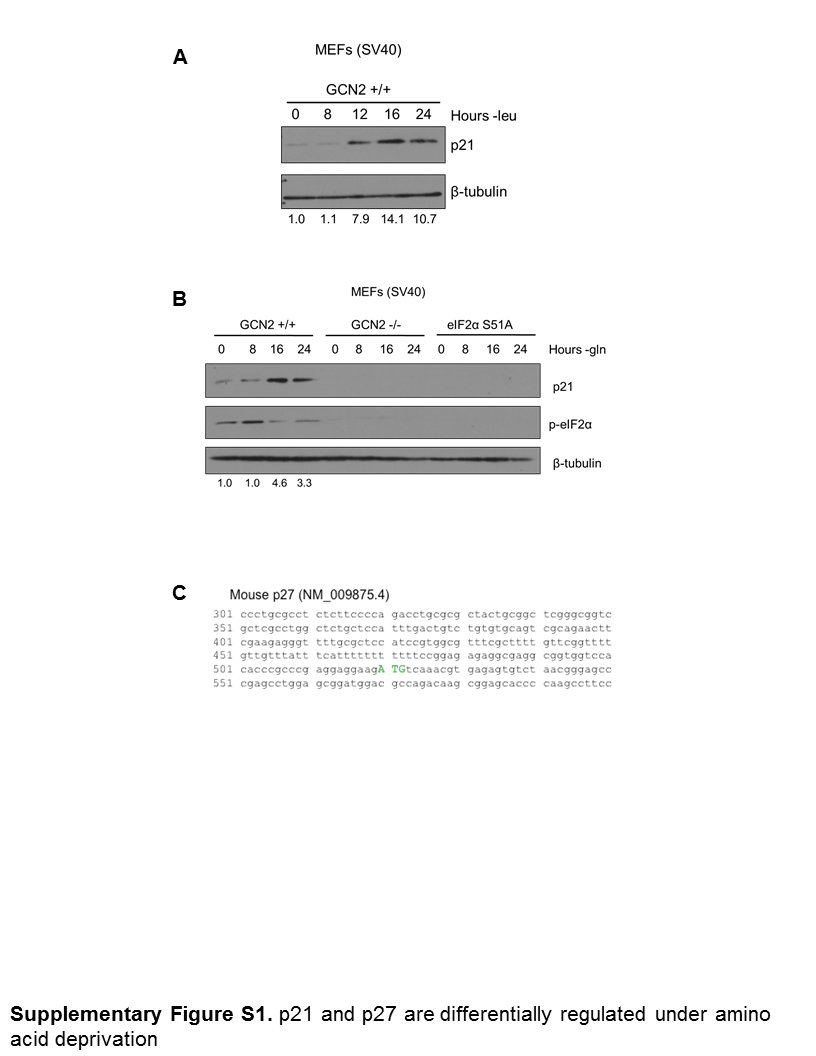

Supplement: S1 Fig — A) Western blot analysis of p21 induction in leucine-deprived GCN2+/+ MEFs. β-tubulin was used as a loading control. Values below blot represent the fold change in total pixel intensity over control of p21 normalized to the loading control for each lane. B) Western blot analysis of p21 induction in glutamine-deprived GCN2+/+, GCN2-/-, and eIF2α S51A MEFs. β-tubulin was used as a loading control. Values below blot represent the fold change in total pixel intensity over control of p21 normalized to the loading control for each lane. C) The 5’ region of mouse p27 mRNA is shown, and the start codon is indicated in green capital letters. There is no upstream start codon present in the 5’ UTR. (TIF) [file pgen.1005212.s001.tif]

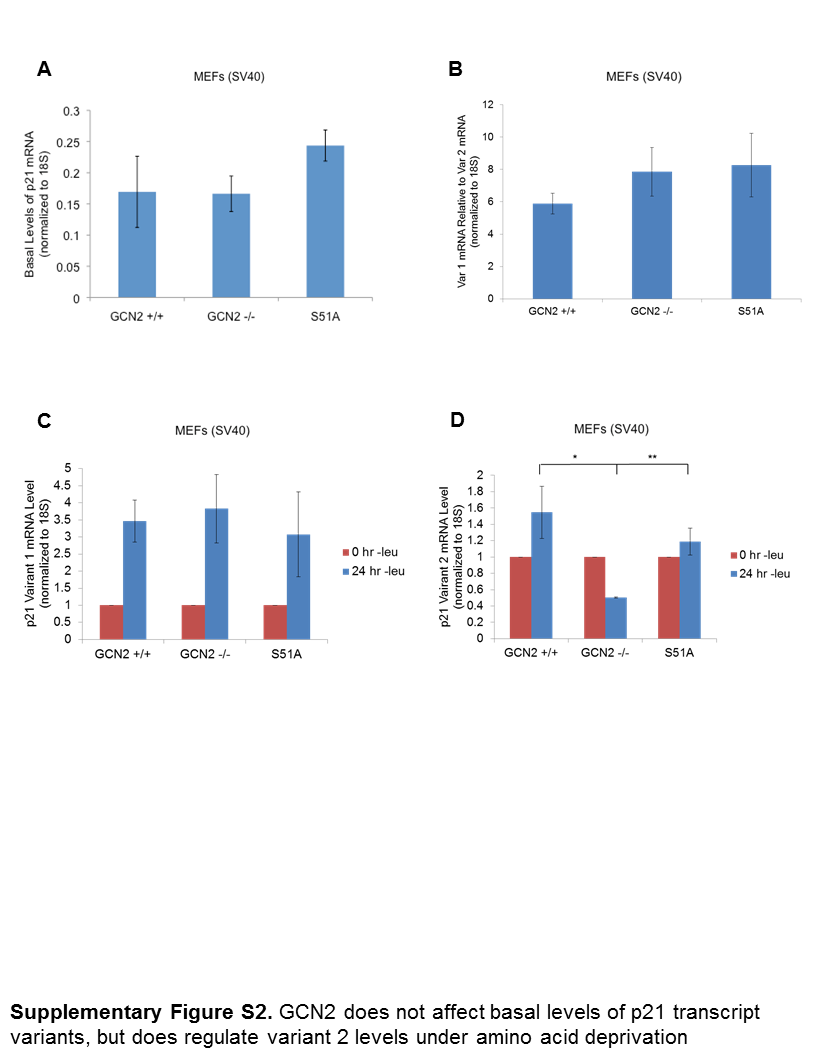

Supplement: S2 Fig — A) qPCR for p21 was performed on RNA isolated from GCN2+/+, GCN2-/-, and eIF2α S51A MEFs grown in complete media. p21 transcript levels were normalized to 18S rRNA. Data represent the average of three independent experiments ± S.E.M. Results were not statistically significant. B) qPCR for p21 variants 1 and 2 was performed on RNA isolated from GCN2+/+, GCN2-/-, and eIF2α S51A MEFs grown in complete media. Levels of transcript variant 1 relative to transcript variant 2 were calculated using the ΔΔCt method using 18S rRNA as the reference gene. Data represent the average of three independent experiments ± S.E.M. Results were not statistically significant. C) qPCR for p21 variant 1 was performed on RNA isolated from GCN2+/+, GCN2-/-, and eIF2α S51A MEFs deprived of leucine for the indicated times. p21 variant 1 transcript levels were normalized to 18S rRNA. Results are depicted as fold change over control for each cell line. Data represent the average of four independent experiments ± S.E.M. There was no statistically significant difference in variant 1 induction among the three cell types. D) qPCR for p21 variant 2 was performed on RNA isolated from GCN2+/+, GCN2-/-, and eIF2α S51A MEFs deprived of leucine for the indicated times. p21 variant 2 transcript levels were normalized to 18S rRNA. Results are depicted as fold change over control for each cell line. Data represent the average of four independent experiments ± S.E.M.; *p<0.05, **p<0.01 (TIF) [file pgen.1005212.s002.tif]

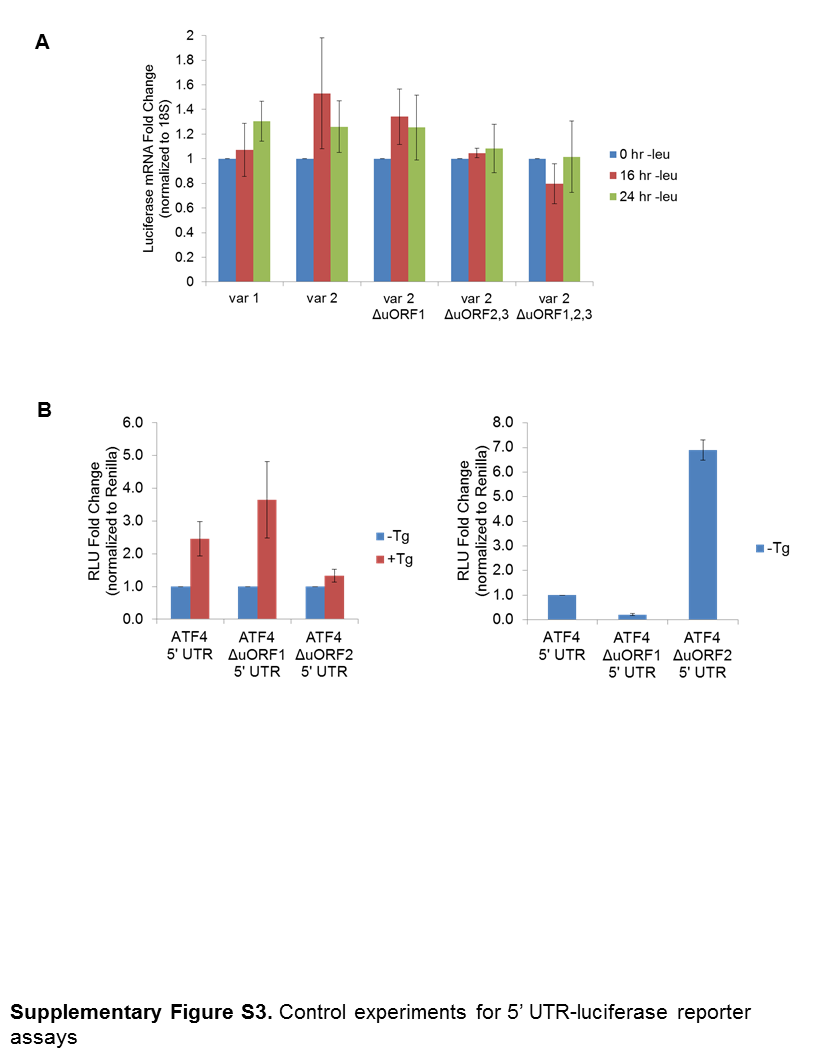

Supplement: S3 Fig — A) qPCR for luciferase was performed on RNA isolated MEFs transfected with the p21 5’ UTR reporter constructs. Luciferase transcript levels were normalized to 18S rRNA and are depicted as fold change over control. Data represent the average of three independent experiments ± S.E.M. No changes in luciferase transcript levels were statistically significant. B) Dual luciferase assay using mutant ATF4 5’ UTR reporter constructs. Left: Luciferase activity from reporter constructs was measured in GCN2+/+ MEFs treated with 1 μM thapsigargin for 6 hours and normalized to Renilla luciferase activity. Right: Relative basal translation levels of ATF4 reporter constructs in untreated cells as measured by luciferase assay. Results are normalized to Renilla and depicted as fold change over the wildtype construct. Data represent the average of three independent experiments ± S.E.M. (TIF) [file pgen.1005212.s003.tif]

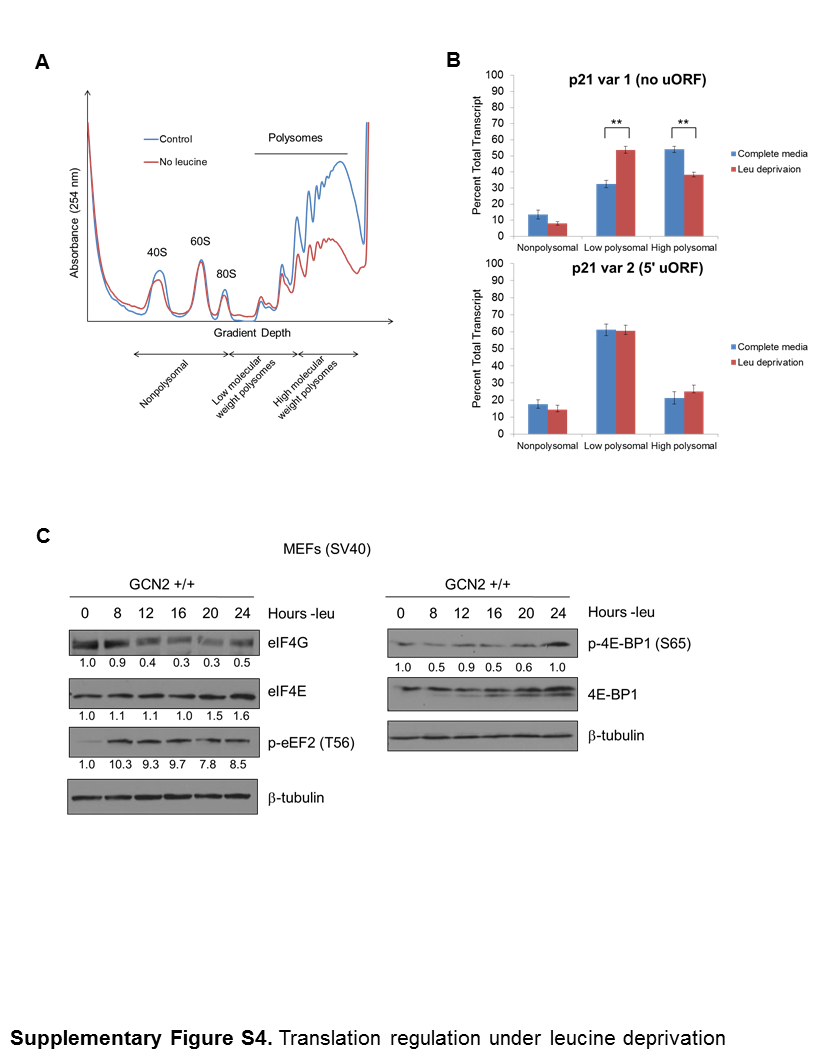

Supplement: S4 Fig — A) Scheme of pooling sucrose gradient fractions into nonpolysomal, low molecular weight polysomal, and high molecular weight polysomal groups for qPCR analysis. This polysome profile is the 24 hour time point from Fig 4A. B) qPCR for both variants of p21 was performed on fractions pooled from sucrose gradients as indicated in (A). p21 transcript levels in each group were normalized to total transcript. Data are the average of three independent experiments ± S.E.M.; **p<0.01. C) Western blot analysis of various components of the translational machinery in leucine-deprived GCN2+/+ MEFs. β-tubulin was used as a loading control. Values below each blot represent the fold change in total pixel intensity over control normalized to β-tubulin (for eIF4G, eIF4E, p-eEF2) or 4E-BP1 (for p-4E-BP1). (TIF) [file pgen.1005212.s004.tif]

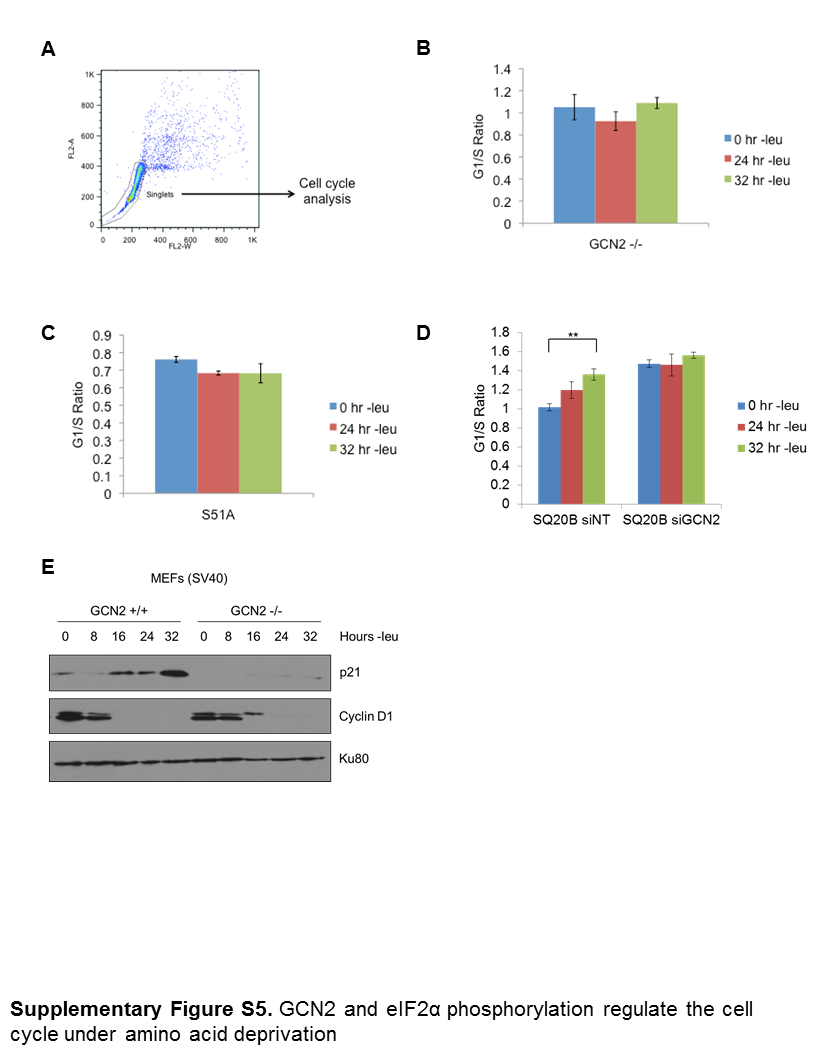

Supplement: S5 Fig — A) Gating strategy used to select single cells for cell cycle analysis by flow cytometry. B) G1/S ratio of GCN2-/- MEFs starved of leucine for the indicated times. DNA content was measured by propidium iodide staining and flow cytometry analysis. Changes in the G1/S ratio were not statistically significant. Data represent the average of three independent experiments ± S.E.M. C) G1/S ratio of eIF2α S51A MEFs starved of leucine for the indicated times. DNA content was measured by propidium iodide staining and flow cytometry analysis. Changes in the G1/S ratio were not statistically significant. Data represent the average of three independent experiments ± S.E.M. D) G1/S ratio of leucine-starved SQ20Bs transfected with non-targeting siRNA (siNT) or siRNA against GCN2 (siGCN2). DNA content was measured by propidium iodide staining and flow cytometry analysis. The knockdown efficiency of siGCN2 is demonstrated in Fig 1E. Data represent the average of three independent experiments ± S.E.M.; ** p<0.01. E) Western blot analysis of cyclin D1 in leucine-starved GCN2+/+ and GCN2-/- MEFs. Ku80 was used as a loading control. (TIF) [file pgen.1005212.s005.tif]

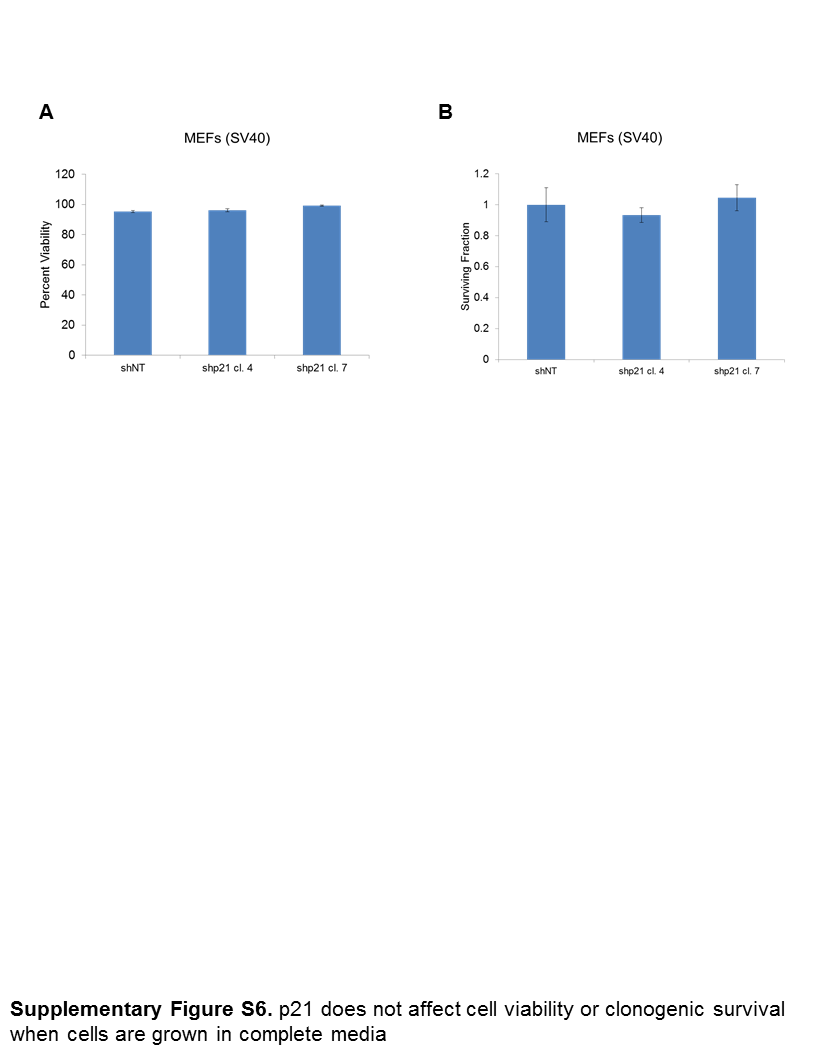

Supplement: S6 Fig — A) Viability of shNT, shp21 cl. 4, and shp21 cl. 7 MEFs grown in complete media as measured by trypan blue exclusion. There was no statistically significant difference in viability between shNT and shp21 cells. Data represent the average of three independent experiments ± S.E.M. B) Clonogenic survival of shNT, shp21 cl. 4, and shp21 cl. 7 MEFs grown in complete media. There was no statistically significant difference in survival between shNT and shp21 cells. Data represent the average of three independent experiments ± S.E.M. (TIF) [file pgen.1005212.s006.tif]
