# Supplementary material for: Translational Upregulation of an Individual p21Cip1 Transcript Variant by GCN2 Regulates Cell Proliferation and Survival under Nutrient Stress
Source: PLoS Genet. 2015 Jun 23;11(6):e1005212. doi: 10.1371/journal.pgen.1005212 (PMC4477940; doi:10.1371/journal.pgen.1005212)
Supplement: S1 Table — (DOCX) [file pgen.1005212.s007.docx]

**Supplementary Table S1. Primer sequences used for qPCR analysis.**

| **Primer Name** | **Sequence (5’ to 3’)** |
| --- | --- |
| *18S* forward | CAATTACAGGGCCTCGAAAG |
| *18S* reverse | AAACGGCTACCACATCCAAG |
| *ATF4* forward | CCTGAACAGCGAAGTGTTGG |
| *ATF4* reverse | TGGAGAACCCATGAGGTTTCAA |
| *β-actin* forward | GGCTGTATTCCCCTCCATCG |
| *β-actin* reverse | CCAGTTGGTAACAATGCCATGT |
| *p21* total forward | CGAGAACGGTGGAACTTTGAC |
| *p21* total reverse | CCAGGGCTCAGGTAGACCTT |
| *p21* var 1 forward | TCCAGACATTCAGAGCCACAG |
| *p21* var 1 reverse | ACGGGACCGAAGAGACAAC |
| *p21* var 2 forward | AGGAGGAGCATGAATGGAGAC |
| *p21* var 2 reverse | GGACATCACCAGGATTGGAC |
